# Adenylate Cyclase Type III Is Not a Ubiquitous Marker for All Primary Cilia during Development

**DOI:** 10.1371/journal.pone.0170756

**Published:** 2017-01-25

**Authors:** Maria Cristina Antal, Karelle Bénardais, Brigitte Samama, Cyril Auger, Valérie Schini-Kerth, Said Ghandour, Nelly Boehm

**Affiliations:** 1 Institut d'Histologie, Service Central de Microscopie Electronique, Faculté de Médecine, Université de Strasbourg, Strasbourg, France; 2 Fédération de Médecine Translationnelle de Strasbourg (FMTS), Strasbourg, France; 3 Hôpitaux Universitaires de Strasbourg, Strasbourg, France; 4 ICube laboratory UMR 7357, team IMIS, Strasbourg, France; 5 UMR CNRS 7213, Laboratoire de Biophotonique et Pharmacologie, Faculté de Pharmacie, Université de Strasbourg, Illkirch, France; 6 Anatomy and Neurobiology, VCU, Richmond, VA, United States of America; National Cancer Institute, UNITED STATES

## Abstract

Adenylate cyclase type III (AC3) is localized in plasma membrane of neuronal primary cilium and can be used as a marker of this cilium. AC3 has also been detected in some other primary cilia such as those of fibroblasts, synoviocytes or astrocytes. Despite the presence of a cilium in almost all cell types, we show that AC3 is not a common marker of all primary cilia of different human and mouse tissues during development. In peripheral organs, AC3 is present mainly in primary cilia in cells of the mesenchymal lineage (fibroblasts, chondroblasts, osteoblasts-osteocytes, odontoblasts, muscle cells and endothelial cells). In epithelia, the apical cilium of renal and pancreatic tubules and of ductal plate in liver is AC3-negative whereas the cilium of basal cells of stratified epithelia is AC3-positive. Using fibroblasts cell culture, we show that AC3 appears at the plasma membrane of the primary cilium as soon as this organelle develops. The functional significance of AC3 localization at the cilium membrane in some cells but not others has to be investigated in relationship with cell physiology and expression at the cilium plasma membrane of specific upstream receptors.

## Introduction

Most differentiated mammalian cells extend a primary cilium. The axoneme of the cilium is made of nine peripheral microtubule doublets (9 + 0) but lacks the two central tubules present in motile cilia. The cilium grows from the mature mother centriole of the diplosome of non-proliferating cells [1, 2]. Primary cilia have chemosensory or mecanosensory functions, according to the specific signaling pathways addressed to the cilium. Pathways implicated in environmental cues detection through membrane receptors have been documented in some cell types including receptor-dependent pathways such as Sonic Hedgehog or non-canonical-Wnt pathways [3, 4], somatostatin receptor 3 [5], serotonin receptor 6 [6], melanin-concentrating hormone receptor 1 [7], dopamine receptor 1 [8], PDGF receptor [9] or extracellular matrix receptors [10].

It has been shown that downstream of ciliary G-protein-coupled receptor (GPCR) signaling, adenylate cyclase type III (AC3) is at present the only effector clearly identified. AC3 is a membrane-bound G-protein regulated adenylyl cyclase which is highly expressed in the olfactory cilia where odorant receptor activation leads to a transduction cascade involving the G-protein alpha subunit, followed by activation of AC3 and the cAMP-dependent opening of calcium channels [11, 12, 13]. In addition, Berbari et al. [14] and Bishop et al. [15] showed that AC3 specifically localizes to almost all neuronal primary cilia in adult mouse brain and AC3 has been detected in some cilia such as those of fibroblasts or synoviocytes [16, 17].

Because of increasing importance of the primary cilium involvement in many biological processes during development, we studied mouse and human embryonic/fetal development of peripheral organs. We here report that AC3 is not a common component of all primary cilia of different human and mouse tissues during development.

## Materials and Methods

### Tissue preparation

#### Animal tissues

All aspects of animal care and the specific experimental procedure for this study were approved by the regional ethics committee (authorization CREMEAS (Comité Régional d’Ethique en Matière d’Expérimentation Animale de Strasbourg) n° AL/41/48/02/13). Mice embryos were removed by caesarian section of timed C57BL/6 pregnant mice, after deep pentobarbital (Vetoquinol, Lure, France) anesthesia of the mother. The whole embryos were fixed in Bouin-Holland fixative. The day of observation of a copulatory plug was considered as gestation day 0 (E0). Three embryos from stages E13, 14, 15, 17 were studied. Adult mice (P60) were perfused transcardially with 4% paraformaldehyde (PFA); the nasal cavities were decalcified in 15% EDTA and embedded in paraffin, as well as the brain and the testis. Five μm paraffin sections from all specimens were cut.

Embryos were serially cut and every 200 microns, adjacent sections were stained with haematoxilin and eosin or immunolabelled for acetylated tubulin or AC3 detection.

Left anterior descending porcine coronary arteries (obtained from the local slaughterhouse) were cleaned of connective tissues and cut into rings which were either used for endothelial cell culture or were fixed in Bouin-Holland fixative and embedded in paraffin.

#### Human tissues

Eight human embryos and fetuses from legal abortion were studied. These embryos/fetuses were collected following requirements and regulations approved by the Medical Ethics Committee of the Faculty of Medicine of Strasbourg for this study. Written informed maternal consents for this study were obtained by an independent physician according to the procedure approved by the ethics committee. The developmental stages were as follows: Carnegie stage (CS) CS 17: 1 embryo; CS 20–21: 2; CS 23: 3; gestational week (GW) 8: 1; GW 12: 1. They were fixed in Bouin-Holland (7 embryo/fetus) or formaldehyde (1) fixative and embedded in paraffin.

### Cell cultures

#### Fibroblast culture

Mouse embryonic fibroblasts (3T3 cell line; Cell culture service, Institut de Génétique, Biologie Moléculaire et Cellulaire, Strasbourg, France) were grown in cell culture chamber slides containing Dulbecco’s Modified Eagle Medium (DMEM) (Gibco, Invitrogen, France) supplemented with 10% calf serum, penicillin (50 U ml^-1^) and streptomycin (50 μg ml^-1^) in an incubator at 37°C with 5% CO_2_. Cells were seeded to obtain increasing levels of cell densities. After fixation and double immunocytochemistry for acetylated-tubulin and AC3, ten fields at x 20 magnification were analyzed for each culture. The number of cilia with each marker was counted and expressed as a percentage of cilia per 100 cells. Each culture was replicated three fold and the results were expressed as the mean of percentage.

#### Endothelial cell culture

Porcine coronary artery endothelial cells were cultured as previously described [18]. Coronary artery segments were flushed with phosphate buffered saline solution (PBS) without calcium to remove remaining blood. Then, endothelial cells were isolated by collagenase type1 treatment (1 mg/ml for 12 min at 37°C) and cultured in flasks containing MDCB131 medium supplemented with 15% fetal calf serum, penicillin (100 U/ml), streptomycin (100 U/ml), fungizone (250 μg/ml), and glutamine (2 mM). Cells were cultured for 48–72 h and confluent cultures were used at first passage.

All cultures used for immunocytochemistry were fixed for 12h with freshly prepared 4% formaldehyde in 0.1 M phosphate buffer pH 7.4.

### Immunocytochemistry

Primary antibodies used were rabbit polyclonal antibodies against AC3 (C20) (1/1000, Santa Cruz Biotechnology, Tebu, Le Perray en Yvelines, France), gamma-tubulin (1/1000, Sigma, Saint Quentin, France), insulin (1/200, Cell Signaling, Danvers, USA) and mouse monoclonal antibodies against acetylated tubulin (1/5000, Sigma, Saint Quentin, France), gamma-tubulin (1/1000, Sigma, Saint Quentin, France), glucagon (1/5000, Sigma, Saint-Quentin, France). For paraffin sections, microwave antigen demasking was followed by overnight incubation in the primary antibody. Incubation with a secondary biotinylated anti-mouse or anti-rabbit antibody was followed by peroxidase-labelled streptavidin complex (Vectastain Elite kit, Vector Laboratories, Abcys, Paris, France); VIP (Vector Laboratories, Abcys, Paris, France) was used as a chromogen and sections or cultures were counterstained with methyl green.

For double immunofluorescence with different species antibodies, sections or cultures were incubated with the primary antibodies overnight. Appropriate secondary antibodies, anti-rabbit Alexa fluor 488 or Alexa fluor 568-labelled and anti-mouse Alexa fluor 568 or Alexa fluor-488-labelled antibodies (1/400, Molecular Probes, Invitrogen, Cergy Pontoise, France) were used. Fluorescence-labelled sections or cultures were conterstained with Dapi (Vectashield, Vector Laboratories, Abcys, Paris, France).

### Statistical analysis

Statistical analysis was performed using two-way analysis of variance followed by a Tukey *post hoc* test for cilia immunocytochemistry in fibroblasts.

## Results

### AC3-positive cilia in mouse adult tissues

**Fig 1:**
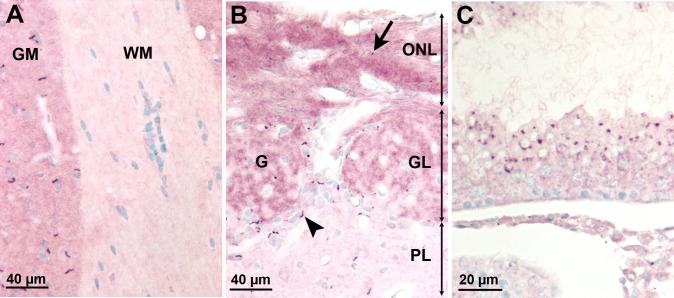
AC3 expression in adult mouse cilia. (A) Brain: note the long AC3 positive cilia in grey matter and the absence of AC3 positive cilia in white matter (corpus callosum). (B) Peripheral layers of the olfactory bulb: note the short AC3-positive cilia in the olfactory nerve layer (arrow) as compared to the long cilia of cells around the olfactory glomeruli (arrow head). (C) Seminiferous tubule: AC3 is present in the developing acrosome of round spermatids. Abbreviations: GM, grey matter; WM, white matter; ONL, olfactory nerve layer; G, glomerulus; GL, glomerular layer; PL, plexiform layer.

We used adult mice brain, olfactory mucosa and testis to assess the accuracy and the specificity of AC3 immunostaining. As previously reported [14, 15], AC3-positive cilia were numerous in the brain cortical and subcortical areas but scattered in white matter (Fig 1A). In the olfactory areas, AC3-positive cilia were present at the tip of olfactory epithelial cells. Cells in the olfactory nerve fascicles in the lamina propria and in olfactory nerve layer of the bulb (ensheathing cells, fibroblasts and astrocytes) had AC3-positive cilia (Fig 1B). Also, AC3 was present in cilia of mitral/tufted, granular and periglomerular cells of the olfactory bulb (Fig 1B). In testis, AC3 was present in round and elongated spermatids as small dots corresponding to developing acrosomes (Fig 1C) and in acrosomes of mature spermatozoa.

### AC3-positive cilia in human and mouse developing organs

**Fig 2:**
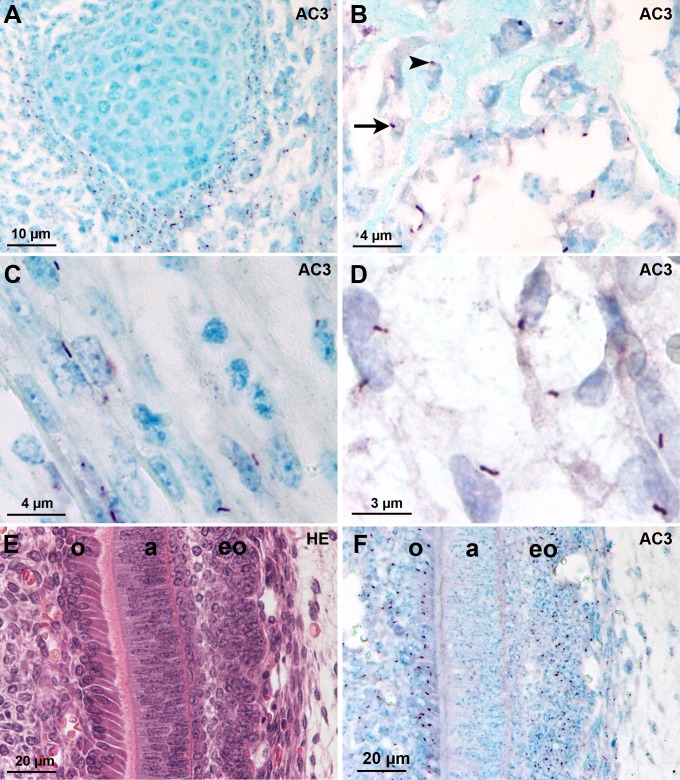
AC3-positive cilia in human and mouse tissues of mesenchymal origin during development. (A-D) Tissue sections from a GW12 human fetus. (A) Meckel’s cartilage: short AC3-positive cilia in perichondral cells and chondroblasts. (B) Bone trabecula: AC3-positive cilia are present in osteoblasts (arrow) and osteocytes (arrow head). (C) Myotubes. (D) Mesenchymal cells. (E, F) Adjacent sections of a mouse tooth at the bell stage. (E) Hematoxylin-eosin staining. (F) AC3 immunohistochemistry shows positive cilia on odontoblasts (o) and peripheral cells of the enamel organ (eo) but not in ameloblasts (a). Abbreviations: AC3: adenylate cyclase type III; HE: Hematoxylin-eosin.

Similar results were observed in human (Table 1) and mouse (Table 2) embryos during development. The main cells exhibiting AC3-positive cilia were of mesenchymal origin: perichondral cells, chondroblasts but not mature and hypertrophic chondrocytes of endochondral ossification (Fig 2A), osteoblasts and osteocytes (Fig 2B), myoblasts and myotubes (Fig 2C), smooth muscle cells of hollow organs, endothelial cells and mesenchymal cells disseminated through the whole body (Fig 2D). Odontoblasts but not differientiated ameloblasts were bearing AC3-positive cilia (Fig 2E and 2F).

**Table 1:** AC3 detection in primary cilia of human developing organs.

			CS17	CS 20–21	CS 23–24	GW8	GW12
**Head structures**	Olfactory epithelium		/	+	+	+	+
** **	Respiratory epithelium	Basal cells	/	/	-	+	+
** **	Tong	Epithelium basal cells	/	/	+	+	+
** **		Muscle cells	/	+	+	+	+
** **		Mesenchymal cells	+	+	+	+	+
** **	Teeth cap stage	Dental papilla	/	/	/	/	+
** **		Enamel epithelium	/	/	/	/	+
** **	Mandibular bone	Osteoblasts	/	+	+	+	+
** **		Osteocytes	/	+	+	+	+
** **	Cartilage	Chondroblasts	/	+	+	+	+
** **		Hypertrophic chondrocytes	/	/	-	-	-
** **		Perichondral cells	/	+	+	+	+
**Kidney**	Metanephrotic blastema		/	+	+	+	+
	Vesicle/S-shaped bodies		/	-	-	-	-
** **	Renal glomerulus	Podocytes	/	/	-	-	-
** **		Bowman capsule	/	/	-	-	-
** **		Mesangial/endothelial cells	/	/	+	+	+
** **	Tubules	Epithelial cells	-	-	-	-	-
** **	Interstitium		+	+	+	+	+
**Gut **		Smooth muscle cells	/	+	+	+	+
** **		Lamina propria	-	+	+	+	+
** Heart**		Myocardium	-	-	-	-	-
**Liver**	Ductal plate	Epithelial cells	/	/	/	-	-
	Periportal space	Mesenchyme	/	/	/	+	+
**Pancreas**		Duct cells	/	-	-	-	-
		Interstitial cells	/	+	+	+	+

/: differentiated cells or structures not yet present; -: AC3 negative cilia; +: AC3 positive cilia.

**Table 2:** AC3 detection in primary cilia of mouse developing organs.

			E13	E14	E15	E17
**Head structures**	Olfactory epithelium		+	+	+	+
** **	Respiratory epithelium	Basal cells	/	+	+	+
** **	Tong	Epithelial basal cells	/	+	+	+
** **		Muscle cells	+	+	+	+
** **		Mesenchymal cells	+	+	+	+
** **	Teeth cap stage	Dental papilla	/	+	+	+
** **		Enamel epithelium	/	+	+	+
** **	Teeth bell stage	Odontoblasts	/	/	/	+
** **		Ameloblasts	/	/	/	-
** **		External epithelium	/	/	/	+
** **	Mandibular bone	Osteoblasts	+	+	+	+
** **		Osteocytes	+	+	+	+
** **	Cartilage	Chondroblasts	+	+	+	+
** **		Hypertrophic chondrocytes	-	-	-	-
** **		Perichondral cells	+	+	+	+
**Kidney**	Metanephrotic blastema		+	+	+	+
** **	Tubules	Epithelial cells	-	-	-	-
** **	Interstitium	Mesenchymal cells	+	+	+	+
**Gut**	Muscle layers	Smooth muscle cells	+	+	+	+
** **	Lamina propria	Mesenchyme	+	+	+	+
**Heart**		Myocardium	-	-	-	-
**Liver**	Intra-hepatic ducts	Epithelial cells	/	-	-	-
	Periportal space	Mesenchyme	/	+	+	+
**Pancreas**	Ducts	Epithelial cells	-	-	-	-
	Interstitium	Mesenchyme	+	+	+	+

/: differentiated cells or structures not yet present; -: AC3 negative cilia; +: AC3 positive cilia.

**Fig 3:**
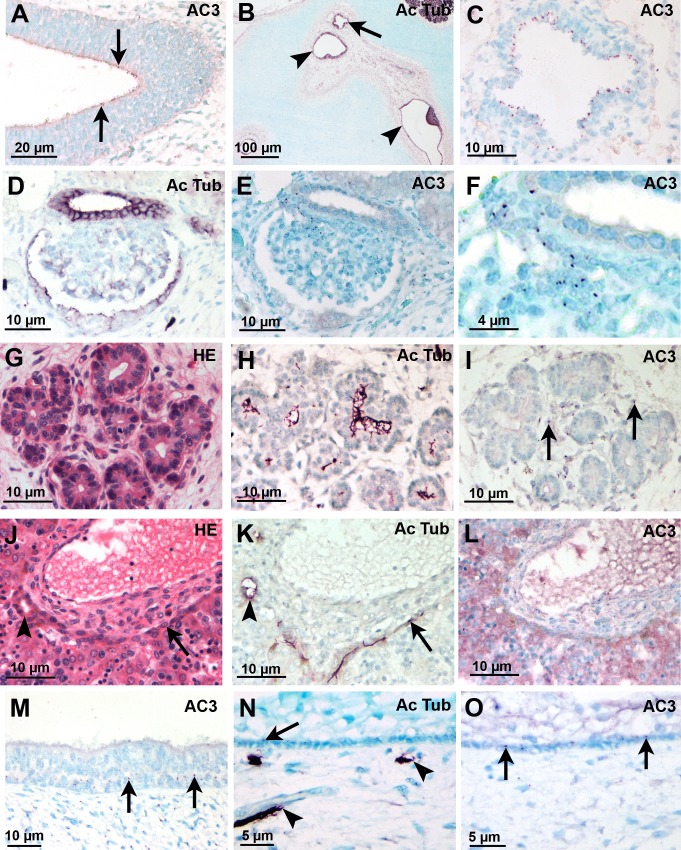
AC3-positive cilia in human epithelia. (A) Olfactory epithelium in GW8 embryo; AC3-positive cilia on olfactory neurons (arrows). (B-C) Inner ear of a GW8 embryo. (B) Section through the endolymphatic duct (arrow) and semicircular ducts (arrow head). (C) Adjacent section showing AC3-positive primary cilia of apical epithelial cells lining the endolymphatic duct. (D-F) Kidney of a GW12 fetus. (D) Primary cilia are present on epithelial cells of the glomerulus and the distal tubule as well as on mesenchymal cells of the floculus and in interstitial cells. (E-F) AC3 is absent from apical epithelial cilia but present in mesenchymal cells cilia of the glomerular floculus and in the interstitium (F is a high magnification of E). (G-I) Pancreas of a GW12 fetus. (G) Hematoxylin-eosin stained section; only ducts but not secretory acini are present at this developmental stage. (H) Apical cilia of pancreatic duct are very long as compared to interstitial cells cilia. (I) Only cilia of cells around the ducts are AC3-positive (arrows). (J-L) liver of a GW12 fetus. (J) Portal space surrounded by the ductal plate (arrow) and small biliary ducts (arrow head). (K) Ductal plate cells (arrow) and the small biliary ducts cells (arrow head) have long primary cilia. (L) Absence of AC3 in epithelial cells cilium. (M-O) Stratified epithelia (M) Respiratory epithelium AC3 positive cilia of basal cells (arrows). (N-O) Oral epithelium. (N) Cilia in basal cells (arrow); presence of small nerve fibers in the lamina propria (arrow heads). (O) The cilia of basal cells are AC3 positive. Abbreviations: AC3: adenylate cyclase type III; Ac Tub: acetylated-tubulin; HE: Hematoxylin-eosin.

AC3 was present at the tip of olfactory neurons (Fig 3A) early in development. In the developing ear, sensory epithelial cells possess AC3-negative kinetocilium and only cells of the endolymphatic duct showed AC3-positive cilia (Fig 3B and 3C).

Apical primary cilia of simple epithelia such as podocytes and external Bowman capsule of the renal glomerulus (Fig 3D–3F), renal tubules (Fig 3D–3F), pancreatic ducts (Fig 3G–3I) and ductal plate (whose remodeling will give rise to intra-hepatic biliary ducts) in the liver (Fig 3J–3L) were AC3-negative. However, mesenchymal cells, either in metanephrotic blastema or in the mesenchyme between the tubes of the kidney (Fig 3E) or in the floculus of the renal glomerulus (Fig 3F), in the mesenchyme between pancreatic ducts (Fig 3I) and in periportal spaces (Fig 3L) had AC3-positive cilia. In the pancreas, cells bearing AC3-positive cilia were not insulin or glucagon endocrine cells. Some AC3-positive cilia were present in basal cells of stratified epithelia (Fig 3M–3O). No acetylated-tubulin and AC3-positive apical cilia were present on intestinal epithelial cells, but they were present in villous lamina propria and deeper mesenchymal and muscular tissues. In the brain and spinal cord, no neuroblasts or glial cells had an AC3-positive cilium.

In both, mouse and human tissues presenting AC3-positive cilia, the number increased with age.

### AC3 expression in fibroblast and endothelial cells in culture

**Fig 4:**
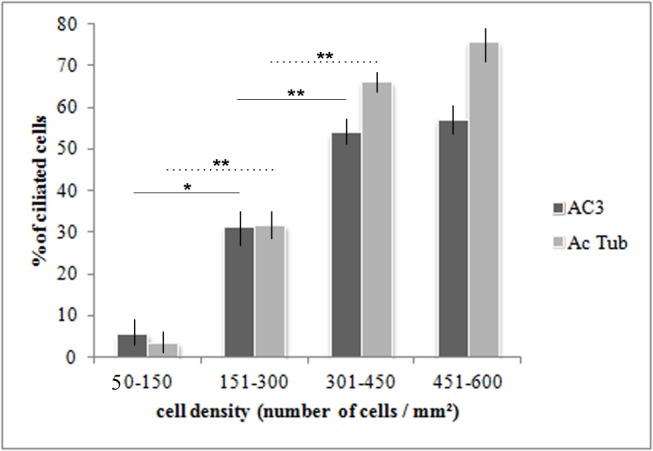
AC3-positive cilia in fibroblast culture. There is an increasing number of fibroblasts extending an AC3 positive cilium with increasing cell confluence. Values represent means ± SEM (*p<0.05; **p<0.01). Solid lines concern AC3 and dotted lines concern acetylated tubulin. Abbreviations: AC3: adenylate cyclase type III; Ac Tub: acetylated-tubulin.

To evaluate the temporal relationship between primary cilium formation and AC3 localization in its membrane, we analyzed fibroblasts in culture. We observed that the percentage of cells bearing a cilium increased with the progression of cell confluence and that the presence of AC3 was correlated with the appearance of the cilium (Fig 4). No difference between AC3 and acetylated tubulin expression was observed.

**Fig 5:**
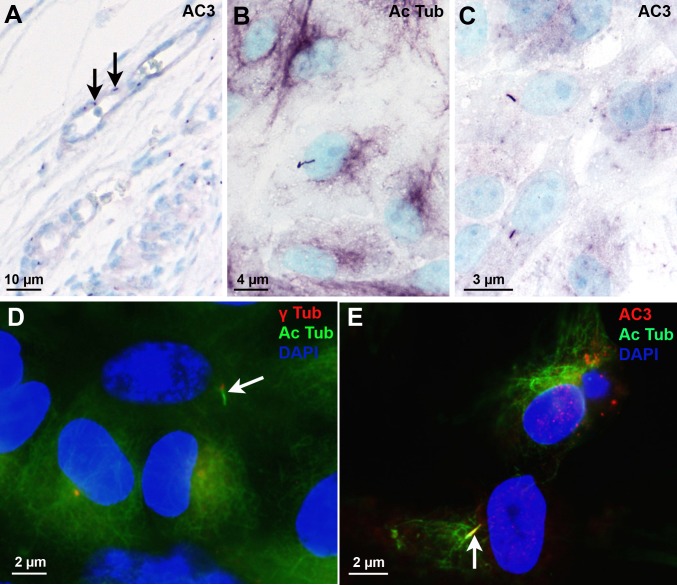
Endothelial cells cilia. (A) Short AC3-positive cilia (arrows) on some endothelial cells in a GW8 human embryo. (B-E) Endothelial cells culture. (B) One cell displays an acetylated tubulin-positive primary cilium. (C) Three adjacent cells display an AC3-positive cilium. (D) One cell extends a primary cilium (arrow) from the gamma tubulin-labelled centrosome. (E) Only one of the three cells displays a primary cilium which is AC3 positive (arrow). Abbreviations: AC3: adenylate cyclase type III; Ac Tub: acetylated-tubulin; γTub: gamma tubulin.

A limited number of endothelial cells were bearing an AC3-positive cilium during development as it was observed *in vivo* (Fig 5A). In primary coronary endothelial cell culture at confluence, few cells had a primary cilium (Fig 5B and 5D), but AC3 expression in these cilia was robust (Fig 5C and 5E). The observation of histological sections of the porcine primary carotids used for endothelial cell cultures showed that very few endothelial cells displayed a cilium.

## Discussion

In different human and mouse tissues, we show that not all primary cilia express the adenylate cyclase type III during development. Adenylate cyclase is the enzyme responsible for synthesis of cAMP from ATP. AC3 was known to be localized to cilia or flagellar structures in adult cells and was first localized to olfactory neurons where it plays a role in the transduction cascade of the olfactory signal through olfactory cilia [11, 12, 13] and in targeting sensory axonal projection to olfactory bulb [19]. Here, we found an early AC3 expression at the tip of the olfactory dendrite in human and mouse, concomitant with the first acetylated-tubulin expression in olfactory cilia.

### AC3 in mesenchymal cells primary cilia

AC3 was mainly present in primary cilia of the mesenchymal cells lineage during development. The primary cilium in mammals is generated during growth arrest and we showed an increase in cilia number with increasing the cell confluence in culture, in agreement with previous reports [20, 21]. In fibroblast culture, we observed that the cilium extension was associated with AC3 expression. This observation is in line with the observation *in vivo*, where tissues of mesenchymal origin displayed increasing number of AC3-positive cilia with the progression of fetal age. It is reasonable then to speculate that AC3 may be used in the signaling pathways activated by blood driven or paracrine ligands binding to membrane receptors. Indeed, some membrane receptors of GPCRs family, localized to the primary cilium, in fibroblasts, were found to be signaling through primary cilia via PDGFR [9].

Insight on the relevance of AC3 presence in cilia was gained from AC3^-/-^ transgenic mice. These mice suffer from anosmia, impaired maternal behavior, cognitive failures, are subfertile and obese with age [12, 13, 19, 22, 23, 24, 25]. However, no other perturbations of peripheral organs such as vessels, bone or cartilage were detected in absence of AC3 in AC3^-/-^ mice. As long as knock-out transgenic mice are concerned, one may hypothesize that during development, other forms of adenylate cyclase may supply for the loss of AC3. In that respect, AC2 and AC4 are expressed in the olfactory cilia of AC3^-/-^ mice [12]. Another hypothesis may be that AC3 is only important during regenerative processes in some mesenchymal cells. An argument for this hypothesis comes also from the olfactory epithelium where AC3-positive cilium of adult basal olfactory progenitors controls olfactory epithelium regeneration without affecting the maintenance of the adult olfactory epithelium [26]. A third hypothesis may be proposed; obesity in AC3^-/-^ mice and protection against obesity in mice with an AC3 gain of function mutation have been attributed to the primary cilium of hypothalamic neurons [23, 27]. Although mature adipocytes do not have a primary cilium, a primary cilium is observed in preadipocytes [28, 29, 30] (the embryos we observed had no identifiable preadipocytes); thus, one cannot exclude that AC3 present in mesenchymal cells cilium could be involved in adipocyte differentiation and resistance to diet-induced obesity.

### AC3 in epithelial cells primary cilia

Many differentiated cells of single layered epithelia have a primary cilium except in the small intestine and colon where enterocytes and goblet cells lack an apical cilium, as demonstrated in the adult mouse [31]. In the present study, we did not identify a primary cilium using either acetylated-tubulin or AC3-directed antibodies during development of these epithelia in mouse and human gut neither at the apical membrane of differentiated enterocytes or goblet cells, nor in cells of the stem cells region during development. We did not observe cilia in the gastric epithelium as did Saqui-Salces et al. [31] in gastric endocrine cells in adult mice; however, it is known that endocrine cells appear after 14W in human [32] and in late fetal period in rodents [33].

A consistent result obtained was the absence of AC3 in apical cilia of epithelial cells. Pancreatic, biliary and renal development is known to be dependent on normal ciliary function. Loss of function or mutation of some proteins in the ciliary membrane or of proteins involved in cilium building lead to renal, biliary and pancreatic cystogenesis in human and mouse [34, 35, 36]. As noted previously, mice lacking AC3 have no reported kidney, pancreas or liver malformation, in accordance with our results. However, we cannot exclude that all primary cilia could mediate cell responses via cAMP production using other types of adenylate cyclases.

AC3 was present in primary cilia of basal cells of stratified epithelia. It has been previously suggested that primary cilia exert a negative control on cell cycle [37, 38, 39]. It has to be noted that AC3-positive cilia have been observed in adult basal olfactory progenitors [40] that may control olfactory epithelium regeneration without affecting the maintenance of the adult olfactory epithelium. No modifications of stratified epithelia have been reported in AC3^-/-^ mice; one can however not exclude a role during regeneration following injury, as was observed for olfactory epithelium.

One epithelial cell type shifted from an AC3 positive to negative cilium during differentiation. Ameloblasts derive from the oral epithelium and have a primary cilium as long they are present. The teeth we could study were incisors and our result are in keeping with the observations of Hisamoto et al. [40] who showed a rapid decline of AC3 expression in the cilium of differentiated molar ameloblasts. Loss of AC3 in ameloblasts cilium could be a sign of functional shift from a ligand dependence to independence via receptor function and AMPc production.

The variability of AC3 presence in primary cilium according to cell phenotype as well as the modulation of AC3 presence by cell differentiation highlight the need to find the membrane receptors each cell type addresses to the cilium membrane to control ubiquitous or specific cell functions.
